# Determinants and protective associations of the lupus low disease activity state in a prospective Chinese cohort

**DOI:** 10.1007/s10067-021-05940-z

**Published:** 2021-09-30

**Authors:** Yanjie Hao, Shereen Oon, Lanlan Ji, Dai Gao, Yong Fan, Yan Geng, Xiaohui Zhang, Guangtao Li, Eric F. Morand, Mandana Nikpour, ZhuoLi Zhang

**Affiliations:** 1 Department of Rheumatology and Clinical Immunology, Peking University First Hospital, 8 Xishiku St, Beijing, 100034 People’s Republic of China; 2 The University of Melbourne Department of Medicine at St. Vincent’s Hospital Melbourne, 41 Victoria Parade, Fitzroy, VIC 3065 Australia; 3 Department of Rheumatology, St. Vincent’s Hospital Melbourne, 41 Victoria Parade, Fitzroy, VIC 3065 Australia; 4 Monash University, Faculty of Medicine, Nursing and Health Sciences, Melbourne, Australia

**Keywords:** Damage accrual, Disease flare, Low disease activity, Systemic lupus erythematosus, Treat-to-target

## Abstract

**Objective:**

To investigate the frequency and determinants of achieving the lupus low disease activity state (LLDAS), and the effect of LLDAS attainment on disease flare and damage accrual in a prospective, single-center cohort of Chinese lupus patients.

**Methods:**

Baseline and follow-up data from consecutive patients at the Peking University First Hospital were collected from January 2017 to June 2020.

**Results:**

A total of 185 patients were enrolled, with median (range) disease duration at enrolment of 2.3 (0.8–7.7) years, and median follow-up of 2.2 (1.0–2.9) years. By the end of the study, 139 (75.1%) patients had achieved LLDAS at least once; 82 (44.3%) patients achieved LLDAS for ≥ 50% of observations. Multivariable logistic regression analysis showed that 24-h urinary total protein (UTP; per g) (OR = 0.447, 95%CI [0.207–0.968], *p* = 0.041), serum creatinine (Scr; per 10 µmol/L) (OR = 0.72, 95%CI [0.52–0.99], *p* = 0.040), and C3 level (per 100 mg/L) (OR = 1.60, 95%CI [1.18–2.17], *p* = 0.003) at recruitment had independent negative associations with achieving LLDAS for ≥ 50% of observations. Kaplan–Meier analyses showed a significant reduction in flare rate with increased proportion of time in LLDAS. Attainment of LLDAS in at least 50% of observations was an independent protective factor for damage accrual (OR = 0.19, 95%CI [0.04–0.99], *p* = 0.049).

**Conclusions:**

In this prospective Chinese cohort, LLDAS was an attainable goal in clinical practice. Nephritis-related markers (UTP and Scr) and C3 level at recruitment negatively influenced achievement of LLDAS. LLDAS achievement was significantly protective from flare and damage accrual.

**Key points:**

*• Low disease activity status (LLDAS) is an achievable target during SLE treatment in China. Urine protein, serum creatinine, and C3 level at recruitment independently affect LLDAS achievement in this group of Chinese lupus patients.*

*• As a treatment target, LLDAS achievement has a highly protective effect for preventing flare and damage accrual, especially in case of achieving LLDAS for ≥ 50% of observations.*

*• The present results further highlight the practical significance of treat-to-target principle in SLE management (T2T/SLE) and the needs for promoting the application of T2T/SLE in clinical practice as well as exploring the concrete implement strategy.*

**Supplementary Information:**

The online version contains supplementary material available at 10.1007/s10067-021-05940-z.

## Introduction

Systemic lupus erythematosus (SLE) is a chronic multisystem autoimmune disease, where treatment is typically long term or even lifelong. With a paucity of advanced therapeutics, most patients are treated with systemic glucocorticoids in addition to immunosuppressants (IS). Despite these, irreversible organ damage and mortality remain unacceptably high. A recent study from the investigators’ center reported that the standardized mortality rate (SMR) of SLE patients in China compared to general population was 3.2 [1]; a meta-analysis of 15 studies comprising 26,101 SLE patients of various ethnicities revealed a similar SMR of 2.7 [2]. Understanding of factors which lead to increased SLE morbidity is therefore paramount.

The principle of treat-to-target (T2T) has been successfully applied to many rheumatological and non-rheumatological diseases. For instance, T2T strategies have dramatically improved the prognosis of patients with rheumatoid arthritis. The concept of T2T in SLE was relatively more recently proposed [3], with several “remission” or “low disease activity” states proposed by an expert task force as treatment targets [4]. The definitions of remission in SLE (DORIS) criteria for remission and lupus low disease activity state (LLDAS) criteria from the Asia–Pacific Lupus Collaboration (APLC) for low disease activity were the most accepted and used definitions [5, 6]. The LLDAS has been both retrospectively and prospectively validated as being associated with reduced damage accrual in several studies [7–9], and found also to be more attainable than remission, whilst being no less protective [10]. With only one prospective validation study previously reported [6], the purpose of the present study was to investigate the frequency and determinants of achieving LLDAS, and the influence of LLDAS on disease flare and damage accrual in a single-center prospective longitudinal cohort of Chinese SLE patients.

## Materials and methods

### Study population

Baseline and follow-up data are prospectively collected from all consecutive patients treated at the Peking University First Hospital in Beijing. Ethics approval was obtained from the ethics committee of the Peking University First Hospital (Project Number: 2017[1284]), and written informed consent obtained from all participants. Subjects fulfilled either the 1997 American College of Rheumatology (ACR) updated classification criteria for SLE [11] or the 2012 Systemic Lupus International Collaborating Clinics (SLICC) classification criteria [12]. No specific treatment algorithm was predefined. Patients were usually followed every 3 months, with 6 months as the maximum allowable interval between consecutive visits. Data from adult SLE patients (≥ 18 years) with at least one follow-up visit between January 2017 and June 2020 were analyzed in this study. Incident patients were defined as the patients who were recruited within 1 year of the onset of their SLE symptoms. Patients were considered lost to follow-up if they had no data recorded for 12 months, and were unable to be contacted after two attempts. The data for these patients collected prior to their loss to follow-up was included in the analysis.

### Data collection

The demographics, disease duration at recruitment, follow-up duration, SLE-related manifestations, and organ involvement, as determined by the ACR classification criteria on an “ever present” basis, were collected. SLEDAI-2K [13], PGA (scale 0–3) [14], and disease flare assessed using the SLE flare index (SFI) [15] were collected at each visit. Irreversible disease damage was captured using the Systemic Lupus International Collaborating Clinics Damage Index (SDI) [16] and health-related quality of life was captured using the short form (36) health survey (SF36) [17], both annually. Damage accrual was defined as an increase of ≥ 1 in SDI. All data were recorded in a standardized electronic case report form as part of the APLC longitudinal cohort study [18]. Of note, data in the present study have not been previously included in APLC publications due to the recency of the Peking University Hospital joining this collaboration. Current use and doses of glucocorticoids, hydroxychloroquine (HCQ) and IS, and laboratory results including complete blood count, renal function, serum albumin (ALB), 24-h urine total protein (UTP), complement levels (C3 and C4), and anti-double stranded DNA (dsDNA) antibody titers at baseline and each follow-up visit were also collected.

### Definitions

Cutaneous and mucosal involvement, leukopenia, thrombocytopenia, and serositis were defined using the 2012 SLICC criteria [12]. Lupus nephritis (LN) was defined as (i) proteinuria > 0.5 grams per day or > 3+, or (ii) cellular casts that may be red cell, hemoglobin, granular, tubular, or mixed, or (iii) biopsy-proven nephritis compatible with SLE [12, 19]. Neuropsychiatric lupus (NPSLE) included a series of disorders ranging from diffuse central nervous system (CNS) disorders (acute confusional state, psychosis, anxiety and depression, and clinical to subclinical cognitive disorders with variable functional significance) to focal CNS syndromes (seizures, cerebrovascular diseases, chorea, myelopathy, transverse myelitis, demyelinating syndrome, aseptic meningitis, headaches) and peripheral nervous system disorders (polyneuropathies, mononeuropathies, autonomic disorders, plexopathy, acute inflammatory demyelination and polyradiculo-neuropathy) [20, 21]. The diagnosis of autoimmune hemolytic anemia (AIHA) was based on evidence of hemolysis including reticulocytosis, bilirubinemia, increased lactate dehydrogenase (LDH), and a positive direct antiglobulin test [22]. Pulmonary arterial hypertension (PAH) was defined as a mean pulmonary artery pressure ≥ 25 mmHg and a pulmonary arterial wedge pressure ≤ 15 mmHg by right heart catheterization, or a mean estimated pulmonary systolic pressure ≥50 mmHg on echocardiography [23].

LLDAS was defined as an SLE disease activity index (SLEDAI)-2 K of ≤ 4, no activity in any major organ system and no features of new disease activity, a physician global assessment (PGA, 0–3) ≤ 1, prednisone dose ≤ 7.5 mg/day, and allowance for maintenance IS and anti-malarials [6].

### Data analysis

Data are reported as mean (standard deviation (SD)) for normally distributed continuous variables, median (interquartile range (IQR)) for skewed continuous data, and percentages or proportions for categorical variables. The Student’s *t*-test was used for comparisons of normally distributed continuous variables, Kruskal–Wallis and Mann–Whitney *U* tests for comparisons of non-normally distributed continuous variables, and the chi-squared test for comparisons of categorical data. Univariate and multivariable logistic regression was used to identify factors associated with achieving LLDAS or damage accrual. Variables with a *p* value ≤ 0.05 in simple logistic regression analyses were included in stepwise multivariable regression analysis. Variables which relate directly to LLDAS assessment such as SLEDAI, PGA, and prednisone dose were not included in the logistic regression model for achieving LLDAS. Flare rate analysis during the follow-up was performed using the Kaplan–Meier method with comparisons performed using the log-rank test. The primary endpoint was disease flare or data censoring. The duration of follow-up was defined as the time from recruitment until the first flare or last follow-up.

All analyses were performed with STATA version 13.1 (StataCorp, College Station, TX, USA) for Windows and a *p*-value of < 0.05 was considered statistically significant.

## Results

### Subject characteristics

Between January 2017 and June 2020, 200 SLE patients were recruited in the prospective cohort. After excluding 15 patients who did not return for follow-up after the first visit, 185 patients including 54 incident patients (29.2%) with total 1203 visits were included in this study. The mean age at disease onset was 33.5 ± 14.9 years with a female predominance (88.1%). The median (IQR) duration at recruitment was 2.3 (0.8–7.7) years, median follow-up duration was 2.2 (1.0–2.9) years, and median number of visits was 7 (4–9). Among the 185 patients, 19 patients were lost to follow-up.

In terms of clinical characteristics, cutaneous and mucosal involvement was most common (61.6% of the patients), followed by LN (55.7%), leukopenia (44.3%), arthritis (39.5%), thrombocytopenia (25.4%), serositis (15.3%), AIHA (9.7%), and NPSLE (8.7%). Other less common disease manifestations included myositis (6.5%), gastrointestinal tract involvement (6.0%), and PAH (4.3%). Renal biopsy was conducted in 62 (33.5%) patients. The median (IQR) SLEDAI-2 K, PGA, and SDI at recruitment were 2 (2–6), 1 (1–2), and 0 (0–1), respectively. One hundred and eighty-two patients (98.4%) received glucocorticoid treatment, 171 (92.4%) received HCQ, and 170 (91.9%) received IS for at least 3 months. During follow-up, 111 (82.1%) patients received IS for at least 6 months and 109 (73.2%) for at least 12 months.

### Frequency of LLDAS attainment

**Fig. 1:**
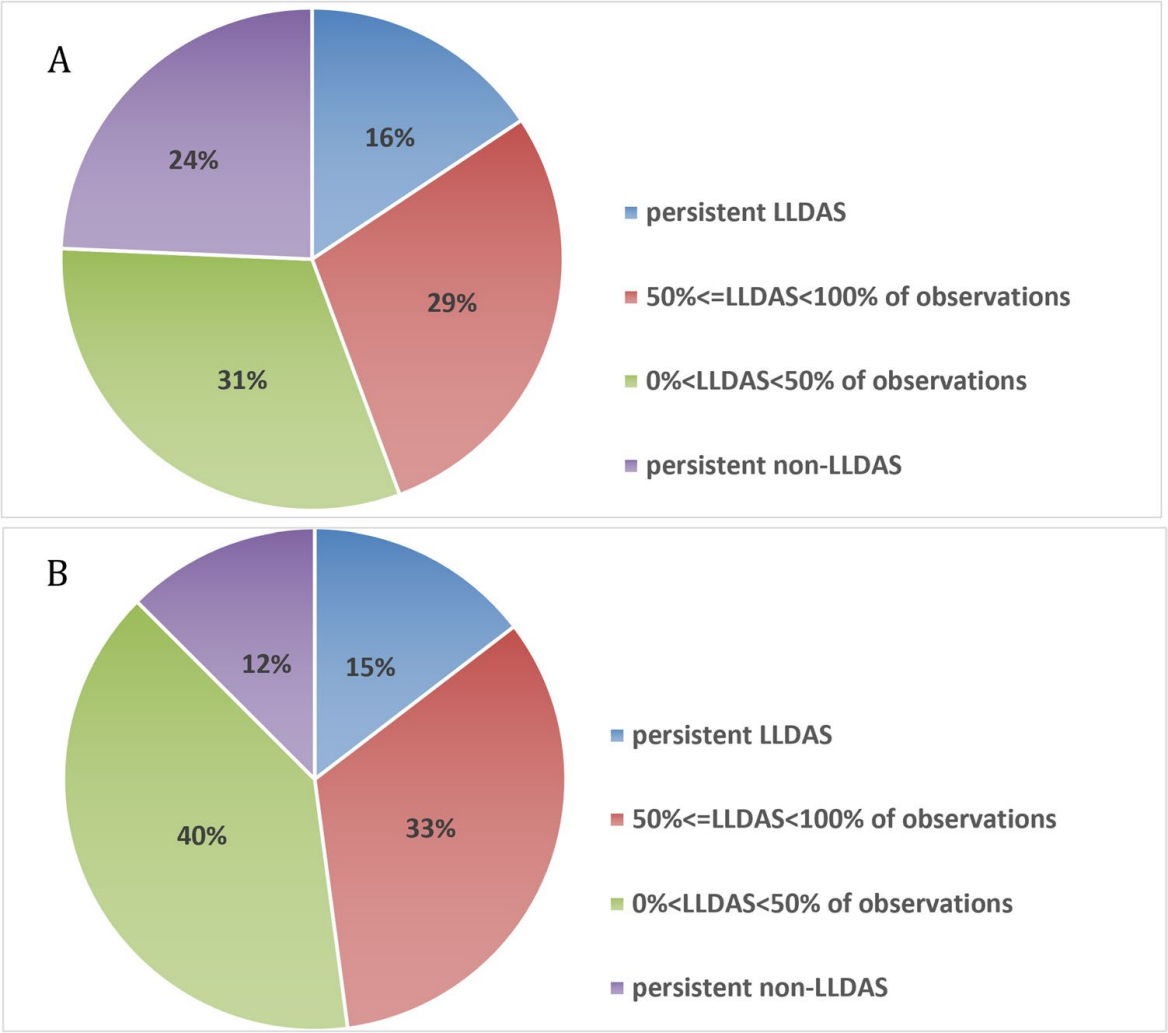
The distribution of percentage observations in LLDAS during follow-up. **A** All patients; **B** patients with at least 12 months of follow-up

In the 185 patients, 58 (31.4%) patients fulfilled LLDAS at recruitment, 81 (43.8%) patients achieved LLDAS during follow-up, and 45 (24.3%) never achieved LLDAS. Therefore, there were 139 (75.1%) patients who achieved LLDAS at least once, including 29 (15.7%) patients who were in LLDAS for 100% of observations, 53 (28.7%) for 50 to 100% of observations, and 58 (31.4%) in LLDAS for < 50% of observations (Fig. 1A). In a subgroup of 144 patients who completed at least 12 months of follow-up, the attainment of LLDAS during the follow-up were shown in Fig. 1B.

### Characteristics of patients achieving LLDAS attainment for ≥ 50% of observations

The characteristics of patients who achieved LLDAS for ≥ 50% of observations were compared to those who did not (Table 1). Compared with subjects who achieved LLDAS for at least 50% of observations (referred to as the LLDAS ≥ 50% group), those who achieved LLDAS for less than 50% of observations (referred to as LLDAS < 50% group) had a higher UTP (390 [50–2300] vs 50 [20–150] mg, *p* < 0.001), lower albumin (39.2 ± 5.3 vs 42.4 ± 5.2 g/L, *p* < 0.001), higher Scr (86.7 ± 34.2 vs 75.6 ± 17.2, *p* = 0.027), lower C3 (714 ± 209 vs 857 ± 200 mg/L, *p* < 0.001), higher SLEDAI-2 K (4 [2–6] vs 2 [0–2], *p* < 0.001), higher PGA (1 [1, 2] vs 0 [0–1], *p* < 0.001), and higher prednisone dose (11.9 ± 3.9 vs 5.4 ± 2.6 mg, *p* < 0.001) at recruitment. The LLDAS < 50% group had a significantly higher proportion of patients who experienced a disease flare during the follow up period (58.6% vs 27.5%, *p* < 0.001) and were more likely to have damage accrual after 2 years of follow-up (48.8% vs 21.4%, *p* = 0.004) than the LLDAS ≥ 50% group. There were no significant differences in individual organ involvement or auto-antibodies between the two groups. Gender, education level, disease duration and SDI at recruitment, and frequency of HCQ and IS use at recruitment and during follow-up were also comparable between the two groups (Table 1).

**Table 1:** Characteristics of SLE patients, categorized by proportion of follow-up observations with achievement of LLDAS

Characteristics	All patients	Non-LLDAS	LLDAS < 50%^Δ^	LLDAS ≥ 50%^Δ^	*P* value ^Δ^
	*n* = 185	*n* = 45	*n* = 58	*n* = 82	
Female, *n* (%)	163 (88.1)	36 (80.0)	50 (86.2)	77 (93.9)	0.122
Education level					
Primary	23 (12.6)	3 (7.0)	9 (15.5)	11 (13.6)	0.946
Secondary	62 (34.1)	14 (32.6)	20 (34.5)	28 (34.6)	
Tertiary	97 (53.3)	26 (60.5)	29 (50.0)	42 (51.9)	
Age at disease onset (years)^†^	33.5 ± 14.9	30.6 ± 13.8	34.3 ± 14.5	34.6 ± 15.8	0.885
Age at recruitment (years)^‡^	39.0 ± 14.9	36.6 ± 13.0	39.2 ± 15.0	40.3 ± 15.7	0.670
Disease duration at recruitment (years)^§^	2.3 (0.8–7.7)	1.2 (0.4–8.3)	2.4 (0.6–7.7)	2.8 (1.3–6.6)	0.660
Duration of follow-up (years)	2.2 (1.0–2.9)	0.7 (0.4–1.2)	2.2 (1.9–2.8)	2.7 (1.6–3.0)	0.242
Organ involvements, *n* (%)^∞^					
Skin & mucous involvement	114 (61.6)	29 (64.4)	38 (66.5)	47 (57.3)	0.328
LN	103 (55.7)	28 (62.2)	33 (56.9)	42 (51.6)	0.556
LN confirmed by biopsy	62 (33.5)	20 (44.4)	19 (32.8)	23 (28.1)	0.171
Leukopenia	82 (44.3)	20 (44.4)	26 (44.8)	36 (43.9)	0.914
Arthritis	73 (39.5)	20 (44.4)	26 (44.8)	27 (32.9)	0.153
Thrombocytopenia	47 (25.4)	11 (21.4)	14 (24.1)	22 (26.8)	0.720
Serositis	28 (15.1)	5 (11.1)	10 (17.5)	13 (16.1)	0.817
NPSLE	16 (8.6)	5 (11.1)	4 (7.0)	7 (8.6)	0.729
AIHA	18 (9.7)	6 (13.3)	7 (12.0)	5 (6.1)	0.214
PAH	8 (4.3)	3 (6.7)	3 (5.2)	2 (2.4)	0.391
Laboratories at recruitment					
Anti-dsDNA positive, *n* (%)	156 (84.3)	38 (84.4)	50 (86.2)	68 (82.9)	0.599
Anti-Sm positive, *n* (%)	52 (28.1)	17 (37.8)	16 (27.6)	19 (23.2)	0.552
C3 (mg/L)	767.2 ± 223.3	667 ± 224	714 ± 209	857 ± 200	< 0.001
C4 (mg/L)	159.5 ± 67.0	136 ± 73	155 ± 62	174 ± 64	0.080
Serum albumin (g/L)	40.4 ± 5.7	38.3 ± 6.0	39.2 ± 5.3	42.4 ± 5.2	< 0.001
UTP (g)	0.15 (0.04–0.95)	0.67 (0.17–1.78)	0.39 (0.05–2.3)	0.05 (0.02–0.15)	< 0.001
Serum Creatinine (μmol/L)	81.6 ± 32.7	86.1 ± 48.2	86.7 ± 34.2	75.6 ± 17.2	0.027
eGFR (mL/min/1.73m^2^)	87.7 (71.9–101.2)	92.0 (76.2–112.0)	87.9 (71.7–94.5)	86.1 (76.9–97.6)	0.754
Scores at recruitment					
SLEDAI-2 K	2 (2–6)	5 (2–10)	4 (2–6)	2 (0–2)	< 0.001
PGA	1 (0–1)	1 (1–2)	1 (1–2)	0 (0–1)	< 0.001
SDI	0 (0–1)	0 (0–1)	0 (0–2)	0 (0–1)	0.311
Treatments					
Prednisone daily dose (mg/d) at recruitment	21.3 ± 17.8	34.6 ± 17.4	27.9 ± 16.7	9.1 ± 8.8	< 0.001
Mean prednisone daily dose (mg/d) during follow-up	11.6 ± 9.2	22.4 ± 11.3	11.9 ± 3.9	5.4 ± 2.6	< 0.001
HCQ, *n* (%)^δ^	171 (92.4)	43 (95.6)	52 (89.7)	76 (92.7)	0.528
Immunosuppressants, *n* (%)					
CTX	29 (15.7)	11 (24.4)	11 (19.0)	7 (8.5)	0.044
MMF	88 (47.6)	20 (44.4)	30 (51.7)	38 (46.3)	0.731
CsA	10 (5.4)	4 (8.9)	3 (5.2)	3 (3.7)	0.458
Tac	6 (3.2)	4 (8.9)	0 (0.0)	2 (2.4)	0.035
AZA	20 (10.8)	5 (11.1)	4 (6.9)	11 (13.4)	0.472
MTX	16 (8.7)	2 (4.4)	7 (12.1)	7 (8.6)	0.395
LEF	21 (11.4)	10 (22.2)	3 (5.2)	8 (9.8)	0.021
Flare during follow-up	73 (40.1)/182	17(38.6)/44	34(58.6)/58	22(27.5)/80	< 0.001
Flare times during follow-up	0 (0–1)	0 (0–1)	1 (0–1)	0 (0–1)	< 0.001
Damage accrual of year 1	21 (14.9)/141	3 (15)/20	11(20)/55	7(10.6)/66	0.150
Damage accrual of year 2	37 (35.6)/104	4(80)/5	21(48.8)/43	12(21.4)/56	0.004

Data are presented as mean ± standard deviation for normally distributed continuous variables, median (IQR) for abnormally distributed continuous variables and numbers (percentages) for categorical variables

Δ non-LLDAS group was defined as patients who had never achieved LLDAS during follow-up, LLDAS < 50% group was defined as patients who achieved LLDAS less than 50% of follow-up time, and LLDAS** ≥ **50% group was defined as patients who achieved LLDAS at least 50% of follow-up time. The comparison was made between LLDAS < 50% and LLDAS** ≥ **50% group

^†^Disease onset defined as the date of first symptom related to SLE

^‡^Recruitment defined as the first date of being recruited in the cohort

^§^Disease duration at recruitment defined as time from disease onset to recruitment

^¶^Duration from recruitment to last visit

^∞^Present ever during course of disease

^δ^Hydroxychloroquine, One IS or the combination of two ISs were used for at least 3 months during follow-up

Abbreviations: *LN* lupus nephritis; *AIHA* autoimmune hemolytic anemia; *NPSLE* neuropsychiatric SLE; *PAH* pulmonary arterial hypertension; *ANA* anti-nuclear antibody; *anti-dsDNA* anti-double-stranded DNA antibody; *C3* complement 3; *C4* complement 4; *UTP* 24-h urine total protein; *eGFR* estimated glomerular filtration rate; *SLEDAI* SLE disease activity index; *PGA* patient global assessment; *SDI* Systemic Lupus International Collaborating Clinics damage index; *HCQ* hydroxychloroquine; *CTX*: cyclophosphamide; *MMF* mycophenolate mofetil; *CsA* cyclosporin; *Tac* tacrolimus; *AZA* azathioprine; *MTX* methotrexate; *LEF* leflunomide

### Determinants of achieving LLDAS for ≥ 50% of observations

Univariate logistic regression analyses showed that higher C3 (per 100 mg/L) (OR = 1.43, 95%CI [1.13–1.74], *p* = 0.000) and Alb (per g/L) (OR = 1.14, 95% CI [1.05–1.24], *p* = 0.001) level at recruitment were positively associated with achieving LLDAS for ≥ 50% of observations. Similarly, higher UTP (per g) (OR = 0.426, 95%CI [0.225–0.805], *p* = 0.009) and Scr (per 10 μmol/L) (OR = 0.79, 95%CI [0.64–0.97], *p* = 0.026) level at recruitment were negatively associated with achieving LLDAS for ≥ 50% of observations. In the multivariable logistic regression model, UTP (per g) (OR = 0.447, 95%CI [0.207–0.968], *p* = 0.041), Scr (per 10 μmol/L) (OR = 0.72, 95%CI [0.52–0.99], *p* = 0.040), and C3 (mg/L) (OR = 1.60, 95%CI [1.18–2.17], *p* = 0.003) at recruitment remained significantly associated with achieving LLDAS for ≥ 50% of observations (Table 2).

**Table 2:** Determinants of achieving LLDAS in at least 50% of observations by univariate and multivariable logistic regression analysis

Variables	Univariate analysis			Multivariable analysis		
	OR	95% CI	*P* value	OR	95% CI	*P* value
Gender: female	2.46	0.76–7.96	0.132			
Education level: tertiary	1.08	0.67–.71	0.760			
Age at disease onset (per year)^†^	1.00	0.98–1.02	0.886			
Disease duration at recruitment (per year)^‡^	1.011	0.96–1.07	0.658			
Follow-up duration (per year)^§^	0.95	0.63–1.45	0.821			
Organ involvements^¶^						
Skin & mucous involvement	0.71	0.35–1.42	0.329			
Arthritis	0.75	0.37–1.50	0.414			
Serositis	1.19	0.27–5.19	0.816			
LN	0.97	0.50–1.91	0.934			
LN confirmed by biopsy	0.54	0.27–1.07	0.076			
NPSLE	1.44	0.25–8.11	0.682			
AIHA	0.44	0.12–1.65	0.226			
Thrombocytopenia	1.47	0.65–3.35	0.358			
Leukopenia	0.79	0.38–1.62	0.513			
PAH	0.46	0.07–2.83	0.401			
Laboratories at recruitment						
Anti-dsDNA positive	0.82	0.41–1.62	0.564			
Anti-Sm positive	0.79	0.37–1.71	0.553			
C3 (per 100 mg/L)	1.43	1.18–1.74	0.000	1.60	1.18–2.17	0.003
C4 (per 100 mg/L)	1.64	0.94–2.88	0.084			
Serum albumin (per g/L)	1.14	1.05–1.24	0.001	1.07	0.94–1.22	0.283
UTP (per g)	0.426	0.225–0.805	0.009	0.447	0.207–0.968	0.041
Serum creatinine (per 10 µmol/L)	0.79	0.64–0.97	0.026	0.72	0.52–0.99	0.040
eGFR (per mL/min/1.73^2^)	0.99	0.98–1.01	0.417			
Scores at recruitment						
SDI	0.80	0.62–1.03	0.079			
Treatments^δ^						
HCQ^δ^	1.46	0.45–4.78	0.530			
CTX ^δ^	0.40	0.14–1.10	0.076			
MMF^δ^	0.81	0.41–1.58	0.530			
CsA^δ^	0.70	0.14–3.58	0.665			
AZA^δ^	2.09	0.63–2.93	0.227			
MTX^δ^	0.69	0.23–2.08	0.510			
LEF^δ^	1.98	0.50–7.82	0.328			

^†^Years; disease onset defined as the date of first symptom related to SLE

^‡^Years; disease duration at recruitment defined as time from disease onset to recruitment

^§^Duration from recruitment to last visit

^¶^Present ever during course of disease

^δ^Hydroxychloroquine or IS was used for at least 3 months during follow-up

Abbreviations: *LN* lupus nephritis; *NPSLE* neuropsychiatric SLE; *AIHA* autoimmune hemolytic anemia; *PAH* pulmonary arterial hypertension; *anti-dsDNA* anti-double-stranded DNA antibody; *C3* complement 3; *UTP* 24-h urine total protein; *eGFR* estimated glomerular filtration rate; *SDI* Systemic Lupus International Collaborating Clinics damage index; *HCQ* hydroxychloroquine; *CTX* cyclophosphamide; *MMF* mycophenolate mofetil; *CsA* cyclosporin; *AZA* azathioprine; *MTX* methotrexate; *LEF* leflunomide

### Protective effect of LLDAS against flare during follow-up

By the end of the study, 73 (39.4%) patients had experienced a disease flare, a mild to moderate flare in 53 (28.6%) patients and severe flare in 20 (10.8%) patients. Fifty-four (23.8%) patients flared within the first 12 months of follow-up and 19 (10.3%) patients after 12 months.

**Fig. 2:**
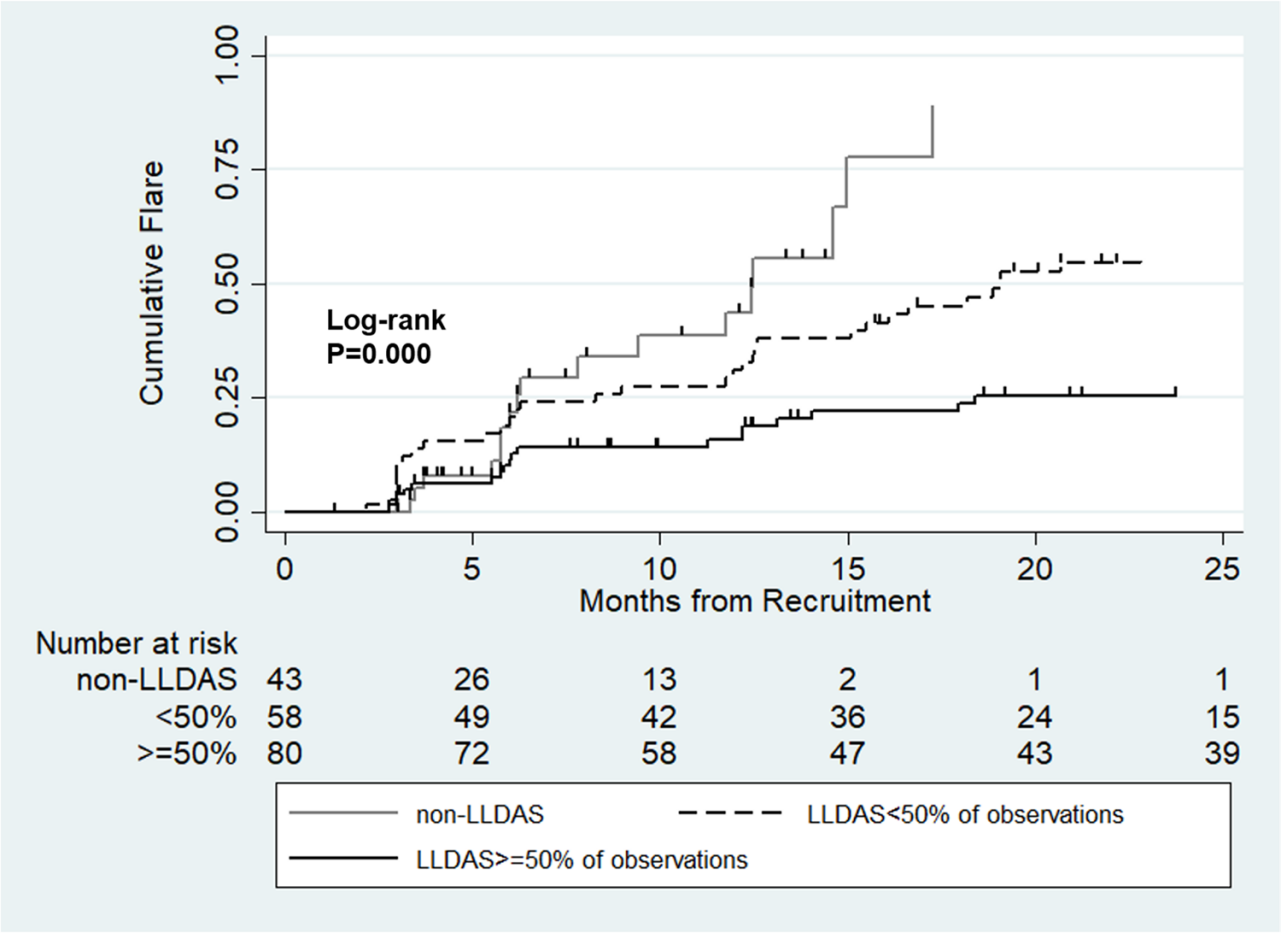
Occurrence of flare curves during follow-up (Kaplan–Meier analysis). The comparison of flare rate was among patients who achieved LLDAS for more than 50% of observations, those who achieved LLDAS less than 50% of observations, and those who never achieved LLDAS (log rank *p* = 0.000)

**Fig. 3:**
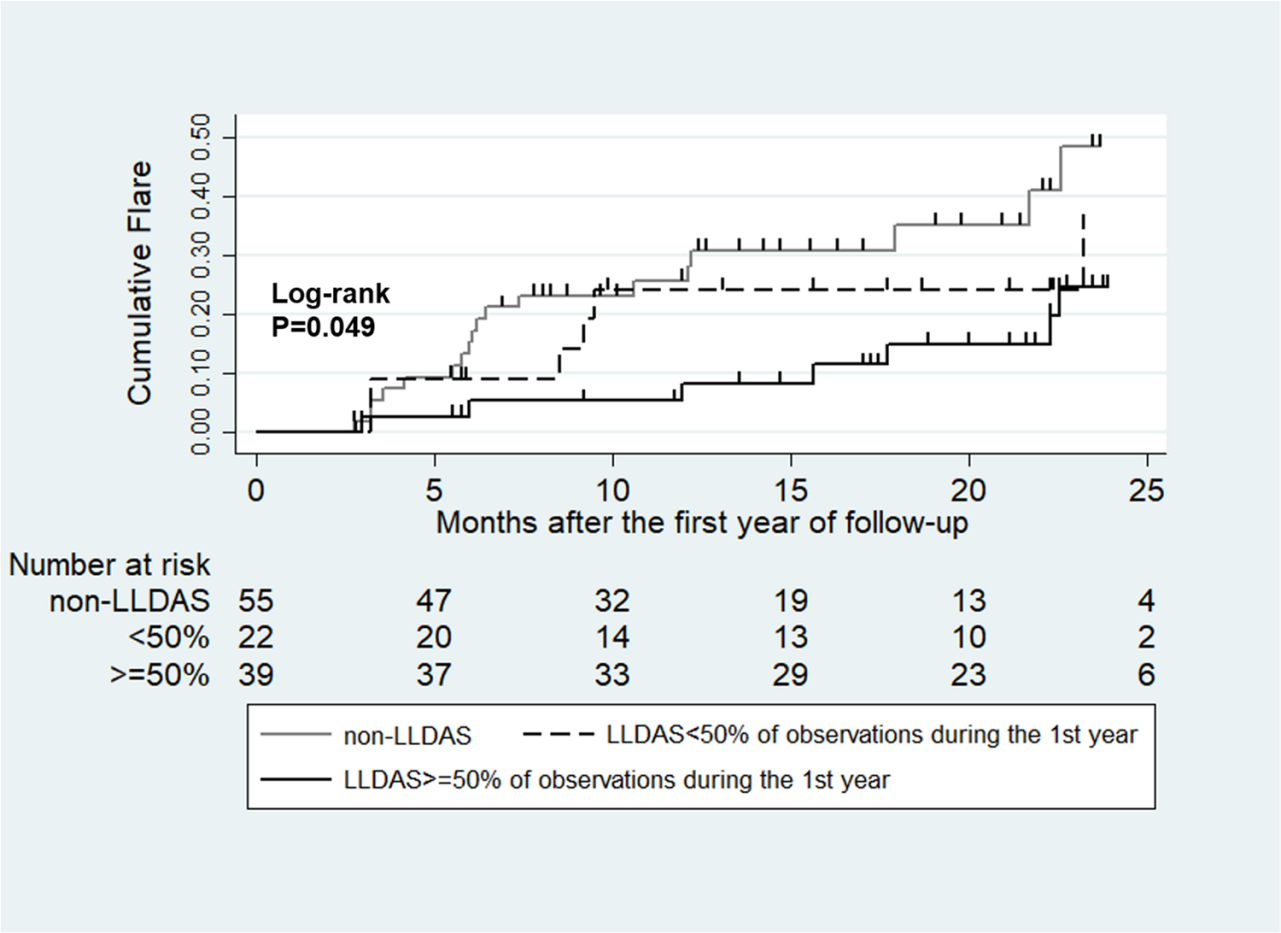
Flare in the second year of follow-up (Kaplan–Meier analysis) in patients categorized by time spent in LLDAS (LLDAS more than 50% of observations, LLDAS less than 50% of observations, and never achieved LLDAS) during the first year of follow-up (log rank *p* = 0.049)

Kaplan–Meier analyses showed that the overall cumulative flare rate was 12% at 6 months, 23% at 12 months, 45% at 24 months, and 52% at 36 months. The flare rate showed a significant difference among patients who never achieved LLDAS, LLDAS < 50%, and LLDAS ≥ 50%, with the lowest flare rate in the LLDAS ≥ 50% group (Log-rank *p* = 0.000, Fig. 2). Meanwhile, the attainment of LLDAS achievement during the first follow-up year was associated with a reduced the risk of flare after the 1st year of follow-up (Log-rank *p* = 0.049, Fig. 3).

### Protective effect of LLDAS on damage accrual during follow-up

The SDI distribution at recruitment and during follow-up is shown in Supplementary Table 1. During follow-up, damage accrual was observed in 21/141 (14.9%) patients within 1 year, 37/104 (35.6%) patients within 2 years, and 11/21 (52.4%) patients within 3 years. In a multivariable logistic regression model, age at disease onset and duration of follow-up (per year) were independent risk factors for damage accrual (OR = 1.05, 95%CI [1.01–1.11], *p* = 0.047; and OR = 6.94, 95%CI [1.89–25.44], *p* = 0.003; respectively), and achieving LLDAS at least 50% of observations during follow-up had a significant protective effect on damage accrual (OR = 0.19, 95%CI [0.04–0.99], *p* = 0.049) (Supplementary Table 2).

## Discussion

LLDAS, an outcome measure developed by the multinational Asia–Pacific Lupus Collaboration, has been validated retrospectively and prospectively to be associated with a reduced risk of organ damage in studies from Latin America, North America, Europe, and the Asia–Pacific region [6, 9, 24]. Here, we present a prospective single-center longitudinal study of LLDAS attainment and its association with disease flare and damage accrual in Chinese patients.

In our cohort, 75.1% of the patients achieved LLDAS at least once and 44.3% were in LLDAS at least 50% of visits, which were comparable with other reports [7, 25] and confirmed that LLDAS is an achievable goal, even in newly diagnosed patients who are more likely to have active disease [26].

Our analyses showed that there were significant differences in baseline characteristics of patients who achieved LLDAS for at least 50% of follow-up observations and those who did not, in terms of baseline UTP, Scr, Alb, and C3. As expected, the variables which are directly involved in the LLDAS assessment criteria such as SLEDAI-2 K, PGA, and prednisone dose were also significantly different between the two groups. Compared with other serological markers such as C4 and anti-dsDNA, baseline C3 was shown in logistic regression analyses to be the most significant marker for subsequent LLDAS attainment. Two previous studies showed that renal involvement was negatively associated with achieving LLDAS [7, 27], but the present study identified that two more specific indicators (UTP and Scr) of renal involvement were independent negative predictive factors for achieving LLDAS** ≥ **50% of observations. Of course, we need to clarify that the patients with LN accounted for more than 50% of all patients in this cohort. In case of without these LN patients, the predicting effect of UTP and Scr would not be statistically significant. A series of epidemiological studies had consistent results with us that nephropathy is one of the most common major organ involvements in SLE patients in China and Asian-Pacific region [1, 7, 28]. The present study further emphasizes the critical role of renal involvement in affecting therapeutic target achievement.

The present study revealed that percentage of time in LLDAS was negatively associated with disease flare. Disease flare is often managed with the use of glucocorticoids and IS, which in turn may lead to further damage accrual or infection risk. A study from the Netherlands showed that having at least one major flare was associated with future damage accrual [25]. The recommendations from the SLE-T2T taskforce underlined that prevention of flares should be a therapeutic goal in SLE and the present study identifies LLDAS as an achievable and important target for preventing flare. These data are encouraging for the future application of the LLDAS in clinical practice as a treatment target.

In the present cohort, we found 14.9% of patients had damage accrual within 1 year, and we observed a significant protective association of LLDAS with damage accrual. Achieving LLDAS ≥ 50% of observations was an independent protective factor for damage accrual. Early damage accrual is a particularly important outcome indicator for SLE patients as it is predictive of further damage accrual [29] and lower survival. For example, one study showed that patients with initial damage had a fourfold higher mortality rate compared to those with no early damage [30]. Another study showed that damage 1 year after diagnosis was a significant predictor of death within 10 years of diagnosis [31].

Interestingly, age of disease onset was found to have different associations with disease flare and damage accrual, wherein older age at disease onset was an independent protective factor for disease flare, while it was an independent risk factor for damage accrual. One study found that patients with younger age at disease onset were more likely to have active disease than those with older age at disease onset [32]. This implies that more vigilant care is needed for these patients. However, consistent with another report [8], older patients were more likely to develop permanent organ damage which need closer monitor and prophylactic treatment.

There are some limitations to this study. First, our sample size was relatively small, the follow-up duration was relatively short especially in those who did not achieve LLDAS, and the loss to follow-up ratio was relatively high, which limits the evaluation for organ damage development and the correlation between LLDAS achievement and damage accrual. Secondly, our assumption that patients are in LLDAS for the entire duration between two visits at which they are found to be in LLDAS may overestimate the duration of being in LLDAS.

In conclusion, LLDAS was an attainable goal in clinical practice in Chinese SLE patients and therefore a potentially applicable goal in a T2T strategy. Higher urine protein and serum creatinine level and lower C3 level at baseline were negative influence factors for achieving LLDAS subsequently. LLDAS is a promising treatment target in SLE, negatively associated with disease flare and damage. Future prospective studies, using large sample sizes, are expected to validate these findings.

## Supplementary Information

Below is the link to the electronic supplementary material.

Supplementary file1 (DOCX 28 kb)

## Data Availability

The datasets analyzed during the current study are available from the corresponding author upon reasonable request.
