# Supplementary material for: Determinants and protective associations of the lupus low disease activity state in a prospective Chinese cohort
Source: Clin Rheumatol. 2021 Sep 30;41(2):357–66. doi: 10.1007/s10067-021-05940-z (PMC8782788; doi:10.1007/s10067-021-05940-z)
Supplement: Supplementary file 1 — Supplementary file1 (DOCX 28 kb) [file 10067_2021_5940_MOESM1_ESM.docx]

**Supplementary table 1–SDI distribution at recruitment and during follow up**

|  | **SDI** | | | | | | | |
| --- | --- | --- | --- | --- | --- | --- | --- | --- |
|  | **0** | **1** | **2** | **3** | **4** | **5** | **6** | **7** |
| **At recruitment** (%)  (n=184) | 64.1 | 13.6 | 9.8 | 4.9 | 4.9 | 1.1 | 1.1 | 0.5 |
| **Year 1** (%)  (n=141) | 56.0 | 18.4 | 7.8 | 8.5 | 5.7 | 1.4 | 1.4 | 0.7 |
| **Year 2** (%)  (n=104) | 47.1 | 19.2 | 14.4 | 7.7 | 6.7 | 1.9 | 1.0 | 1.9 |
| **Year 3** (%)  (n=21) | 38.1 | 23.8 | 9.5 | 14.3 | 9.5 | 4.8 |  |  |

Abbreviations: SDI: Systemic Lupus International Collaborating Clinics damage index.

**Supplementary table 2 –Determinants of damage accrual during 3 years of follow up by univariate and multivariable logistic regression analysis**

| **Variables** | **Univariate analysis** | | | **Multivariable analysis** | | |
| --- | --- | --- | --- | --- | --- | --- |
|  | OR | 95% CI | P value | OR | 95% CI | P value |
| **Gender-Female** | 1.07 | 0.35-3.29 | 0.901 |  |  |  |
| **Education level** | 0.44 | 0.27-0.73 | 0.001 | 0.46 | 0.19-1.11 | 0.085 |
| **Age at disease onset (per year)**^†^ | 1.04 | 1.02-1.07 | 0.001 | 1.05 | 1.01-1.11 | 0.047 |
| **Disease duration at recruitment (per year)**^‡^ | 1.01 | 0.95-1.06 | 0.789 |  |  |  |
| **Duration of follow-up (per year)** ^¶^ | 2.31 | 1.28-4.19 | 0.006 | 6.94 | 1.89-25.44 | 0.003 |
| **Organ involvements**^§^ |  |  |  |  |  |  |
| Skin & mucous involvement | 1.20 | 0.57-2.49 | 0.632 |  |  |  |
| Arthritis | 0.84 | 0.40-1.74 | 0.632 |  |  |  |
| Serositis | 1.01 | 0.38-2.68 | 0.986 |  |  |  |
| LN | 1.80 | 0.86-3.77 | 0.119 |  |  |  |
| LN confirmed by biopsy | 1.27 | 0.61-2.66 | 0.526 |  |  |  |
| NPSLE | 2.81 | 0.88-8.91 | 0.080 |  |  |  |
| AIHA | 1.04 | 0.30-3.63 | 0.956 |  |  |  |
| Thrombocytopenia | 1.17 | 0.53-2.57 | 0.705 |  |  |  |
| Leukopenia | 0.55 | 0.27-1.13 | 0.105 |  |  |  |
| **Laboratories at recruitment** |  |  |  |  |  |  |
| Anti-dsDNA positive | 1.34 | 0.65-2.76 | 0.430 |  |  |  |
| Anti-Sm positive | 1.94 | 0.87-4.29 | 0.104 |  |  |  |
| C3 (per 100 mg/L) | 1.15 | 0.98-1.35 | 0.095 |  |  |  |
| Serum albumin (per g/L) | 0.95 | 0.89-1.01 | 0.118 |  |  |  |
| UTP (per g) | 1.00 | 0.99-1.00 | 0.214 |  |  |  |
| Serum creatinine (per 10µmol/L) | 1.25 | 1.05-1.50 | 0.015 | 1.16 | 0.92-1.48 | 0.211 |
| **Scores at recruitment** |  |  |  |  |  |  |
| SLEDAI | 1.04 | 0.95-1.15 | 0.396 |  |  |  |
| PGA | 1.08 | 0.72-1.63 | 0.702 |  |  |  |
| SDI | 1.55 | 1.19-2.01 | 0.001 | 1.43 | 0.90-2.28 | 0.131 |
| **Treatments** |  |  |  |  |  |  |
| Prednisone daily dose at recruitment (per mg/d) | 1.00 | 0.98-1.02 | 0.765 |  |  |  |
| Cumulative prednisone dose during follow-up (per g) | 1.02 | 1.01-1.03 | 0.015 | 0.999 | 0.998-1.001 | 0.857 |
| HCQ^δ^ | 0.22 | 0.05-0.91 | 0.037 | 0.20 | 0.02-2.06 | 0.177 |
| CTX^δ^ | 4.76 | 1.82-12.41 | 0.001 | 1.15 | 0.20-6.55 | 0.878 |
| MMF^δ^ | 0.58 | 0.29-1.19 | 0.138 |  |  |  |
| CsA^δ^ | 2.92 | 0.63-13.63 | 0.173 |  |  |  |
| AZA^δ^ | 0.39 | 0.08-1.84 | 0.233 |  |  |  |
| MTX^δ^ | 0.67 | 0.17-2.59 | 0.558 |  |  |  |
| LEF^δ^ | 0.81 | 0.24-2.73 | 0.734 |  |  |  |
| **LLDAS≥50% of observations** ^Δ^ | 0.38 | 0.18-0.83 | 0.015 | 0.19 | 0.04-0.99 | 0.049 |

† Years; disease onset defined as the date of first symptom related to SLE.

‡ Years; disease duration at recruitment defined as time from disease onset to recruitment.

§ Present ever during course of disease.

δ Hydroxychloroquine or IS was used for at least three months during follow up.

Δ Achieved LLDAS in at least 50% of observations during the follow-up

Abbreviations: LN: lupus nephritis; NPSLE: neuropsychiatric SLE; AIHA: autoimmune hemolytic anemia; PAH: pulmonary arterial hypertension; anti-dsDNA: anti-double-stranded DNA antibody; C3: complement 3; UTP: 24-hour urine total protein; SLEDAI: systemic lupus erythematosus disease activity index; SDI: Systemic Lupus International Collaborating Clinics damage index; HCQ: Hydroxychloroquine; CTX: Cyclophosphomide; MMF: Mycophenolate Mofetil; CsA: Cyclosporin; AZA: Azathioprine; MTX: Methotrexate; LEF: Leflunomide; LLDAS: lupus low disease activity status.
